# Renal impairment associated with tenofovir disoproxil fumarate for antiretroviral therapy and HIV pre-exposure prophylaxis: An observational cohort study

**DOI:** 10.1371/journal.pone.0280339

**Published:** 2023-02-24

**Authors:** Jack E. Heron, Hamish McManus, Tobias Vickers, Kathleen Ryan, Edwina Wright, Allison Carter, Mark Stoove, Jason Asselin, Andrew Grulich, Basil Donovan, Rebecca Guy, Rick Varma, Marcus Chen, Nathan Ryder, David A. Lewis, David J. Templeton, Catherine C. O’Connor, David M. Gracey, for the ACCESS Collaboration

**Affiliations:** 1 Department of Renal Medicine, Royal Prince Alfred Hospital, Camperdown, New South Wales, Australia; 2 Department of Medicine, Albury Wodonga Health, Wodonga, Victoria, Australia; 3 The Kirby Institute, University of New South Wales, Kensington, New South Wales, Australia; 4 Department of Infectious Disease, Alfred Health, Melbourne, Victoria, Australia; 5 Burnet Institute, Melbourne, Victoria, Australia; 6 Peter Doherty Institute, Melbourne, Victoria, Australia; 7 Sydney Sexual Health Centre, Sydney Hospital, Sydney, New South Wales, Australia; 8 Melbourne Sexual Health Centre, Alfred Health, Melbourne, Victoria, Australia; 9 Central Clinical School, Monash University, Melbourne, Victoria, Australia; 10 Hunter New England Clinic, Tamworth, New South Wales, Australia; 11 School of Medicine and Public Health, University of Newcastle, Callaghan, New South Wales, Australia; 12 Western Sydney Sexual Health Centre, Western Sydney Local Health District, Parramatta, New South Wales, Australia; 13 Faculty of Medicine and Health, Westmead Clinical School, University of Sydney, Westmead, New South Wales, Australia; 14 Marie Bashir Institute for Infectious Diseases and Biosecurity, University of Sydney, Westmead, New South Wales, Australia; 15 Faculty of Medicine and Health, University of Sydney, Camperdown, New South Wales, Australia; 16 Department of Sexual Health Medicine and Sexual Assault Medical Service, Sydney Local Health District, Sydney, New South Wales, Australia; University of Washington, UNITED STATES

## Abstract

**Background:**

Tenofovir disoproxil fumarate (TDF) is associated with adverse renal outcomes when prescribed for HIV infection. There are few data concerning real-world renal outcomes amongst patients prescribed TDF for pre-exposure prophylaxis (PrEP).

**Methods and findings:**

Data were extracted from 52 sexual health clinics across Australia from 2009–2019. All patients prescribed TDF-containing antiretroviral therapy and PrEP were included. Rates of renal impairment (a fall in eGFR to <60 ml/min/1·73m^2^) were calculated for people living with HIV (PLWHIV) prescribed TDF and HIV negative PrEP-users. Risk factors were assessed using Cox-proportional hazards models. Sensitivity analysis of risk using 1:1 propensity-score matching to adjust for potential imbalance in HIV and PrEP cohorts was conducted. 5,973 patients on PrEP and 1,973 PLWHIV were included. There were 39 (0.7%) instances of renal impairment in the PrEP group and 81 (4.1%) in the PLWHIV cohort (hazard ratio [HR]:0.35 95% confidence interval [CI]: 0.22–0.56). Rates of renal impairment were 4.01/1000 person-years (95%CI:2.93–5.48) in the PrEP cohort and 16.18/1000 person-years (95%CI:13.01–20.11) in the PLWHIV cohort (p<0.001). Predictors of renal impairment were: older age (40–49 years (HR:5.09 95%CI: 2.12–12.17) and 50–82 years (HR:13.69 95%CI: 5.92–31.67) (compared with 30–39 years) and baseline eGFR<90ml/min (HR:61.19 95%CI: 19.27–194.30). After adjusting for age and baseline eGFR the rate of renal impairment remained lower in the PrEP cohort (aHR:0.62 95%CI: 0.40–0.94, p = 0.023). In propensity-matched analysis using 1,622 patients per cohort the risk of renal impairment remained higher in the PLWHIV cohort (log-rank p = 0.001).

**Conclusion:**

Patients prescribed TDF-based PrEP had lower rates of renal impairment than patients prescribed TDF for HIV infection. In propensity analysis, after matching for some risk factors, rates of renal impairment remained higher amongst patients with HIV.

## Introduction

Australia has adopted a national goal of ending HIV transmission. To achieve this goal HIV prevention strategies, including pre-exposure prophylaxis (PrEP), have been implemented and rapidly scaled-up. Daily oral PrEP, using fixed dose Tenofovir disoproxil fumarate (TDF) in combination with emtricitabine (FTC), was rolled out in Australia in 2014. Following EPIC-NSW and PrEPX, two large multi-centre prospective PrEP implementation studies, PrEP has been subsidised by the Australian Pharmaceutical Benefits Scheme (PBS) [1,2]. Event driven PrEP was not recommended until 2020, and Tenofovir alafenamide (TAF) based PrEP has not yet been approved in Australia.

Among people living with HIV (PLWHIV), antiretroviral therapy (ART), in particular TDF and ritonavir-boosted regimens are associated with an increased risk of acute kidney injury, proteinuria and chronic kidney disease (CKD) [3,4]. There are few data concerning renal outcomes among patients prescribed TDF for PrEP. Adverse renal events were uncommon in randomised controlled trials of PrEP; however, patients with renal impairment, proteinuria and other risk factors for kidney disease were typically excluded [5–16].

HIV is known to infect renal proximal tubular cells and may potentiate ART toxicity [17]; an effect which could explain the relatively low rate of adverse renal events in PrEP trials. It is not known if patients prescribed PrEP outside the setting of randomised clinical trials are at an increased risk of adverse renal outcomes. In this study we compare the rates of sustained renal impairment, which we define as an eGFR of <60 ml/min on two consecutive measurements at least 30 days apart, between participants receiving ART for HIV infection and participants prescribed PrEP in a ‘real world’ setting.

## Methods

### Study population

We conducted a retrospective longitudinal open cohort study. For a flow diagram describing the enrolment process see Appendix 1. Individuals were included from the first date they had a record of being prescribed TDF for HIV infection or PrEP during attendance at an ACCESS clinic (see below) between 1 January 2009 and 31 December 2019. Patients were included if they had a baseline eGFR (defined as the eGFR measurement recorded at the closest test date to treatment commencement within the interval from up to 180 days prior to treatment until 30 days after treatment commencement) and had at least two further eGFR test results available after treatment commencement. Patients were excluded if they had pre-existing renal impairment defined for the purpose of this study as an eGFR <60 ml/min/1·73m^2^ at or any time prior to baseline. Participants were followed for up to three years from treatment commencement. We defined renal impairment to be an eGFR measurement of less than 60 ml/min/1·73m^2^. PLWHIV were identified as those on a clinic diagnosis register or, who had repeated viral load testing, or were prescribed combination ART. PrEP participants were identified as those patients receiving TDF/FTC without any other ART and excluded those patients prescribed TDF/FTC for post-exposure prophylaxis after 1 January 2015.

### Data source

Data were extracted from 52 clinics that were participating in a sentinel surveillance program known as ACCESS; as previously described [18,19]. The dataset was collected as part of routine care and securely extracted via GRHANITE™ software. Anonymous probabilistic linkage was applied to the dataset and unified patient records across multiple clinics.

### Comorbidities

Comorbid renal impairment, chronic hepatitis B, and chronic or past hepatitis C were identified through medication, laboratory and diagnostic history. Hepatitis B status was based on hepatitis B surface Antigen detection (HBsAg), whereas hepatitis C was identified through ever having hepatitis C antibody (HCV Ab) positivity.

### Statistical analysis

Participants on PrEP with eGFR records were followed from date of first PrEP until either reaching censor date at 31 December 2019 or sustained renal impairment. Sustained renal impairment was defined as the first date recording an eGFR <60 ml/min/1·73m^2^ and confirmed by a subsequent measurement.

Participants were censored during intervals of greater than 365 days without eGFR testing with censoring effective from 182 days after last eGFR measurement.

Participant numbers by descriptive characteristics were tabulated by treatment cohort (PLWHIV, PrEP-users) including: *socio demographic indicators*: age group (18–29 years, 30–39 years, 40–49 years, over 50 years), gender (female, male, trans/other), culturally and linguistically diverse status (yes/no), area of residence (major city, inner regional, outer regional/remote), state or territory (New South Wales (NSW)/Australian Capital Territory (ACT), Victoria, Queensland, South Australia (SA)/Western Australia (WA)/Northern Territory (NT), Tasmania); *behavioural risk factors* men who have sex with men (yes/no), injecting drug use (yes/no); *clinical risk factors* baseline eGFR (60–90, greater than 90 ml/min/1.73m^2^), HBsAg positivity (time updated), and HCV Ab positivity (time updated). Participants with missing data for gender (<0.6%) were assigned ‘male’ status.

Rates of time to sustained renal impairment by cohort and by risk factor above were developed as rates per 1000 person years with differences assessed using log rank tests. Cumulative probability of renal impairment over time was estimated using the Kaplan-Meier method, with difference between survivor curves measured using log rank tests. Univariate Cox proportional hazards models stratified by clinic, were used to assess determinants of time to renal impairment for risk factor listed above. Variables which were significant in univariate analyses at p = 0.05 level were included in a multivariate model to assess the adjusted difference in time to impairment between PLWHIV and PrEP-users and a parsimonious model developed by backward stepwise model selection.

A propensity score matched analysis was then conducted to assess risk difference between cohorts (ART and PrEP) after matching on confounders for treatment cohort. Matching was conducted using propensities from a multivariate logistic regression of baseline covariates above. Matching was based on nearest neighbour within-calliper matching, with callipers set at 0.25 of the propensity score standard deviation [20]. Goodness of logistic model fit was assessed using Hosmer-Lemeshow’s test and overall balance in the model assessed for the full dataset and matched subsample respectively using student’s t-test as well as for separate categorical predictors using Pearson’s chi-squared tests of the standardised difference between cohorts. Differences in time to sustained renal impairment were then assessed as above using log-rank test and the Kaplan-Meier method. A univariate Cox model of fit was also generated from the matched data.

All analyses were conducted using Stata version 15.1, StataCorp, Texas, USA.

### Ethics

Ethics approval for the project was provided by the Human Research Ethics Committees at Alfred Hospital (248/17), Central Australia Human Research Ethics Committee at Flinders University. (CA-19-3355), Northern Territory Department of Health and Menzies School of Health (08/47), University of Tasmania (H0016971), Aboriginal Health and Medical Research Council (1099/15), ACON (2015/14), Victorian AIDS Council / Thorne Harbour Health (VAC REP 15/003), *Western Australian Aboriginal Health Ethics Committee (885)*, and St. Vincent’s Hospital (08/051). As our study analysed de-identified data collected under the auspices of public health surveillance, individual participant consent was not required. Individuals were able to opt-out of the surveillance network.

## Results

7,946 participants were included in the analysis, including 1,973 PLWHIV taking ART and 5,973 PrEP-users. The baseline characteristics of the two cohorts are shown in Table 1. Participants in the PrEP cohort were younger and more likely to be male than the PLWHIV cohort). Renal impairment at baseline was more common among PLWHIV (50.3% versus 25.2%, p<0.001). Past or current hepatitis B infection as evidenced by a previous positive hepatitis B surface antigen was uncommon in both cohorts but occurred statistically more frequently in PLWHIV (0.8% versus 0.6%, p = 0.008). Hepatitis C antibody positivity was tenfold more common in PLWHIV (8·8% versus 0·8%, p<0·001).

**Table 1:** Baseline characteristics.

Characteristic	Category	PLWHIV on ART (%)	PrEP-users (%)
Count		1973 (100·0)	5973 (100·0)
Sustained renal impairment	Yes	77 (3·9)	39 (0·7)
	No	1896 (96·1)	5934 (99·3)
Age group in years	median (IQR)	44 (36–52)	36 (29–46)
	16–29	224 (11·4)	1644 (27·5)
	30–39	473 (24·0)	1936 (32·4)
	40–49	634 (32·1)	1321 (22·1)
	50+	642 (32·5)	1072 (17·9)
Treatment start	2010–2012	786 (39·8)	8 (0·1)
	2013–2015	667 (33·8)	142 (2·4)
	2016–2019	520 (26·4)	5823 (97·5)
Sex	male	1829 (92·7)	5850 (97·9)
	female	143 (7·2)	77 (1·3)
	other		8 (0·1)
	missing/unknown	1 (0·1)	38 (0·6)
CALD	no	1565 (79·3)	4948 (82·8)
	yes	408 (20·7)	1025 (17·2)
Region of birth	Australia	856 (43·4)	2875 (48·1)
	East Asia	202 (10·2)	569 (9·5)
	Europe	113 (5·7)	447 (7·5)
	Middle East/Africa	96 (4·9)	132 (2·2)
	North America	20 (1·0)	91 (1·5)
	Oceania	61 (3·1)	128 (2·1)
	South America	43 (2·2)	155 (2·6)
	Unknown	582 (29·5)	1576 (26·4)
Index eGFR category	<90	992 (50·3)	1502 (25·1)
	≥90	981 (49·7)	4471 (74·9)
MSM	No	468 (23·7)	244 (4·1)
	Yes	1505 (76·3)	5729 (95·9)
Injective drug use	No	1836 (93·1)	5797 (97·1)
	Yes	137 (6·9)	176 (2·9)
HBsAg antibody positive (ever)	No	1954 (99·0)	5952 (99·6)
	Yes	19 (1·0)	21 (0·4)
HCV antibody positive (ever)	No	1799 (91·2)	5927 (99·2)
	Yes	174 (8·8)	46 (0·8)
State	NSW/ACT	1604 (81·3)	4174 (69·9)
	QLD	49 (2·5)	262 (4·4)
	SA/WA/NT	186 (9·4)	466 (7·8)
	TAS	87 (4·4)	132 (2·2)
	VIC	47 (2·4)	939 (15·7)

ACT = Australian Capital Territory, ART = antiretroviral therapy, PrEP = pre-exposure prophylaxis, CALD = culturally and linguistically diverse, eGFR = estimated glomerular filtration rate, HBsAg = hepatitis B surface antigen, HCV = hepatitis C virus, MSM = men who have sex with men, NSW = New South Wales, NT = Northern Territory, PLWHIV = people living with HIV, QLD = Queensland, SA = South Australia, TAS = Tasmania, VIC = Victoria, WA = Western Australia.

### Rates of sustained renal impairment

**Fig 1:**
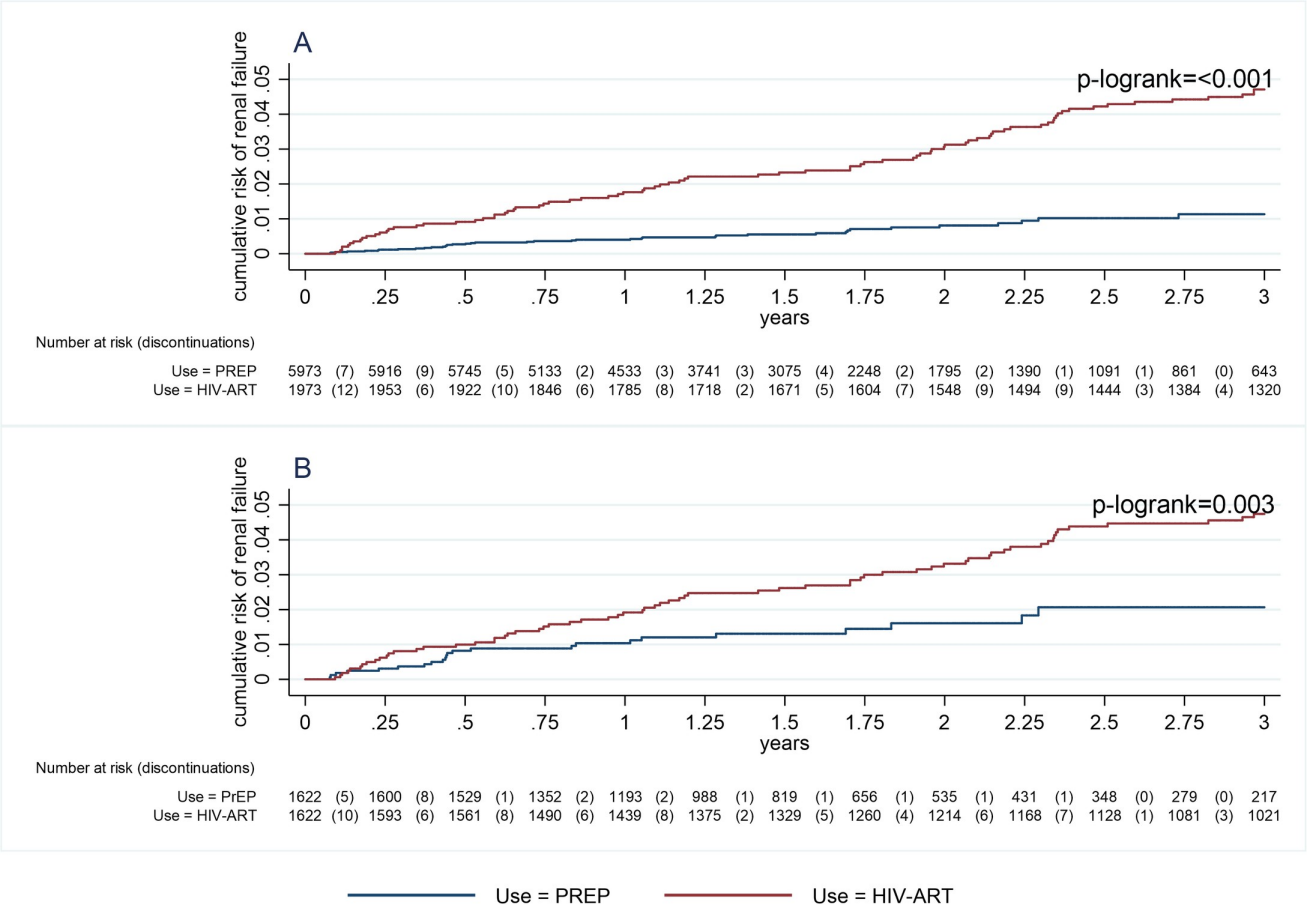
Text: Kaplan-Meier failure curves for time to sustained renal failure for PLWHIV receiving antiretroviral therapy compared to HIV negative participants receiving PrEP before (Panel A) and after (Panel B) propensity score matching).

There were 39 instances of sustained renal impairment in PrEP participants (0·7%) and 77 instances of sustained renal impairment in the PLWHIV (3·9%). Over 9,740 person-years of follow-up among PrEP participants, rates of sustained renal impairment were 4·01/1000 person-years of follow-up (95% confidence interval (CI): 2·93–5·48) (observations for. Over 5,010 person-years of follow-up in HIV-positive participants, rates of sustained renal impairment were significantly higher at) 16·18/1000 person-years of follow-up (95% CI: 13·01–20·11). PLWHIV (observations for). A log-rank test indicated difference in Kaplan Meier survivor functions (p<0·001) with a higher renal impairment rate indicated for PLWHIV receiving ART (Fig 1).

### Predictors of sustained renal impairment

Cox proportional hazards model showed the risk of sustained renal impairment in PrEP participants was 65% lower than amongst PLWHIV receiving ART (HR 0·35 (95% CI 0·22–0·56), p<0·001) (Table 2). Rates of sustained renal impairment were lower in age groups <40 years, when compared to older age groups. In a multivariate model, adjusted for age group and baseline renal impairment, there remained a statistically significant difference in the rate of sustained renal impairment between PrEP participants and PLWHIV (HR 0·62 (95% CI 0·40–0·94), p = 0·023). No interaction effects were detected between PrEP status and other independent predictors (results not shown).

**Table 2:** Cox proportional hazards models of time until sustained renal impairment. Models stratified on testing clinic.

Variable	Variable	HR (95% CI)	Wald p	HR (95% CI)	Wald p
**HIV status**	PrEP use	0·35 (0·22–0·56)	<0·001	0·62 (0·40–0·94)	0·0234
	PLWHIV on ART	1 (ref)	·	1 (ref)	·
			·		
**Age group**	16–29	0·00 (0·00-·)	1·000	0·00 (0·00-·)	1·000
	30–39	1 (ref)	·	1 (ref)	·
	40–49	5·09 (2·12–12·17)	<0.001	3·22 (1·35–7·69)	0·009
	50–82	13·69 (5·92–31·67)	<0·001	6·90 (2·99–15·89)	<0·001
			·		
**Sex**	male	1 (ref)	·		
	female	0·90 (0·33–2·46)	0·836		
			·		
**MSM**	No	0·65 (0·34–1·25)	0·196		
	Yes	1 (ref)	·		
			·		
**Injecting drug use**	No	1 (ref)	·		
	Yes	0·55 (0·13–2·29)	0·411		
			·		
**Baseline eGFR**	<90 ml/min/1·73m^2^	61·19 (19·27–194·30)	<0·001	32·08 (10·11–101·78)	<0·001
	≥90 ml/min/1·73m^2^	1 (ref)	·	1 (ref)	·
			·		
**HBsAg ever (time updated)**	HBsAg negative	1 (ref)	·		
	HBsAg positive	0	1·0000		
			·		
**HCV ever (time updated)**	HCV antibody negative	1 (ref)	·		
	HCV antibody positive	0·97 (0·36–2·66)	0·959		
			·		

Participants were followed for up to three years from treatment commencement. Participants were censored at loss-to-follow-up (365 days without testing) commencing at 180 days from last test, or at study censor date 31 December 2019. Renal impairment defined as date of later test of successive eGFR<60ml/min/1.73m^2^ in tests separated by at least 30 days. Time-updated Hepatitis B antigen status was not included in the model as there were no instances of sustained renal impairment among participants who had prior evidence of Hepatitis B antigen positivity.

PrEP = pre-exposure prophylaxis, PLWHIV = people living with HIV, ART = antiretroviral therapy, eGFR = estimated glomerular filtration rate, HBsAg = hepatitis B surface antigen, HCV = hepatitis C virus, MSM = men who have sex with men.

### Propensity analysis

Participant characteristics before and after propensity score matching are shown in Table 3. Significant imbalance was detected between the PrEP (n = 5,934) and PLWHIV cohorts (n = 1,892) before propensity score matching (p<0.001) with most covariates being unbalanced between cohorts apart from remote residence. After propensity score matching using the variables listed in Table 3, overall imbalance was reduced (p = 0·067) with all covariates balanced except for age group (p = 0·01).

**Table 3:** Comparison of the variables before and after propensity score matching.

		Before matching					After matching				
		PLWHIV		PREP usersPrEP			PLWHIV		PREP usersPrEP		
	Category	n	%	n	%	p	n	%	n	%	p
**Sustained renal impairment**	No	1892	96	5934	99	<0.001	1552	96	1599	99	<0.001
	Yes	81	4	39	1	·	70	4	23	1	·
**Age group in years**	16–29	224	11	1644	28	<0.001	219	14	227	14	0·012
	30–39	473	24	1936	32	·	402	25	375	23	·
	40–49	634	32	1321	22	·	518	32	470	29	·
	50–82	642	33	1072	18	·	483	30	550	34	·
**eGFR**	60–90	992	50	1502	25	<0.001	775	48	797	49	0·440
	90+	981	50	4471	75	·	847	52	825	51	·
**CALD**	No	988	50	3369	56	0·007	842	52	882	54	0·093
	Yes	403	20	1028	17	·	304	19	307	19	·
	Unknown	582	30	1576	26	·	476	29	433	27	·
**HCV (baseline)**	Negative	1875	95	5940	99	<0.001	1583	98	1593	98	0·222
	Positive	98	5	33	1	·	39	2	29	2	·
**HBsAg (baseline)**	Negative	1957	99	5953	100	0·008	1610	99	1610	99	1·000
	Positive	16	1	20	0	·	12	1	12	1	·
**Index Year**	2010–13	1082	55	11	0	<0.001	895	55	8	1	<0.001
	2014–16	500	25	1183	20	·	409	25	384	24	·
	2017–19	391	20	4779	80	·	318	20	1230	76	·
**Sex**	Male	1830	93	5896	99	<0.001	1557	96	1562	96	0·648
	Female	143	7	77	1	·	65	4	60	4	·
**MSM**	No	468	24	244	4	<0·001	174	11	180	11	0·735
	Yes	1505	76	5729	96	·	1448	89	1442	89	·
**Injective drug use (ever)**	No	1836	93	5797	97	<0·001	1507	93	1506	93	0·946
	Yes	137	7	176	3	·	115	7	116	7	·
**State**	NSW/ACT	1604	81	4174	70	<0·001	1334	82	1387	86	0·921
	VIC	47	2	939	16	·	47	3	39	2	·
	QLD	49	3	262	4	·	47	3	51	3	·
	SA/WA/NT	186	9	466	8	·	142	9	92	6	·
	TAS	87	4	132	2	·	52	3	53	3	·

ACT = Australian Capital Territory, ART = antiretroviral therapy, PrEP = pre-exposure prophylaxis, CALD = culturally and linguistically diverse, eGFR = estimated glomerular filtration rate, HBsAg = hepatitis B surface antigen, HCV = hepatitis C virus, MSM = men who have sex with men, NSW = New South Wales, NT = Northern Territory, PLWHIV = people living with HIV, QLD = Queensland, SA = South Australia, TAS = Tasmania, VIC = Victoria, WA = Western Australia.

Using 1,622 pairs of participants obtained by propensity score matching, survival analysis was performed between the PrEP and PLWHIV cohorts. There were 23 instances of sustained renal impairment in PrEP participants (1·42%) and 66 instances of sustained renal impairment in the PLWHIV (4·07%). Rates of sustained renal impairment for three years of follow-up were 8·6/1,000 person years of follow-up (95% CI: 5·7–13·0) in PrEP participants (observations for 2,700 years of follow-up) compared to 16·5/1,000 person years of follow-up (95% CI: 13·0–21·0) in PLWHIV (observations for 4,000 years of follow-up). A log-rank test indicated difference in Kaplan Meier survivor functions (p = 0·003) with higher impairment rate indicated for PLWHIV receiving ART. In a Cox regression analysis the risk of sustained renal impairment amongst PrEP participants was 52% lower than amongst PLWHIV receiving ART (HR 0·48 (95%CI 0·30–0·78), p<0·0031) (Table 4).

**Table 4:** Propensity score analysis. Cox proportional hazards models of time until sustained renal impairment. Models stratified on testing clinic.

		HR (95% CI)	Wald p	HR (95%CI)	Wald p
**Cohort**	PrEP group	0·48 (0·30–0·78)	0·0031	0·53 (0·30–0·94)	0·0297
	PLWHIV	1 (ref)	·	1 (ref)	·
			·		
**Age group**	16–29	0·00 (0·00–0·00)	·	0·00 (0·00-·)	1·0000
	30–39	1 (ref)	·	1 (ref)	·
	40–49	4·47 (1·55–12·92)	0·0057	2·93 (1·01–8·52)	0·0487
	50–82	12·55 (4·57–34·50)	<0·001	6·94 (2·50–19·31)	0·0002
			·		
**Gender**	male	1 (ref)	·		
	female	0·30 (0·04–2·19)	0·2376		
			·		
**MSM**	No	0·21 (0·05–0·85)	0·0288		
	Yes	1 (ref)	·		
			·		
**Injecting drug use**	No	1 (ref)	·		
	Yes	0·14 (0·02–0·99)	0·0490		
			·		
**Index eGFR (ml/min/1.73m**^**2**^)	<90	92·92 (12·94–667·04)	<0·001	51·35 (7·10–371·22)	0·0001
	≥90	1 (ref)	·	1 (ref)	·
			·		
**HCV ever (time updated)**	Negative	1 (ref)	·		
	Positive	0·00 (0·00-·)	1·0000		

ART = antiretroviral therapy, eGFR = estimated glomerular filtration rate, HCV = hepatitis C virus, HR = hazard ratio, MSM = men who have sex with men.

Time-updated Hepatitis B antigen status was not included in the model because of there were no instances of sustained renal impairment among participants who had prior evidence of Hepatitis B antigen positivity.

## Discussion

In this study we found that the risk of sustained renal impairment, defined as new onset of an eGFR <60 ml/min/1·73m^2^ confirmed on repeated testing, was 65% lower among participants prescribed TDF for PrEP compared with those prescribed TDF-based ART for HIV infection. In a propensity score analysis including 1,622 pairs matched for viral hepatitis status and other variables we found that PrEP participants remained at over 50% lower risk of sustained renal impairment compared with those taking TDF/FTC for HIV infection. These real-world data support the safety of TDF-based PrEP by demonstrating a low rate of adverse renal outcomes.

ART prescribed for the treatment of HIV infection is associated with adverse renal outcomes. Among participants with HIV infection, treatment with TDF, in particular, is associated with a reduction in eGFR and progression to chronic kidney disease [21,22]. The reduction in eGFR is most rapid in the first year of treatment [23] with the risk of renal impairment also possibly related to cumulative drug exposure [22]. Renal impairment may be irreversible if TDF is continued in the setting of new renal impairment [24]. A similar and cumulative effect is seen with ritonavir-boosted atazanavir and ritonavir-boosted lopinavir [22]. Renal impairment, defined as an eGFR of between 60 and 90 ml/min/1·73m^2^ appears to be common among participants on TDF-based ART for HIV infection, occurring in one-third of participants [24]. In addition to effects of renal function measured by eGFR, TDF is also associated with renal proximal tubular dysfunction including proteinuria, glycosuria, hypouricaemia and hypophosphataemia [25]. These adverse renal outcomes appear to be associated with older age, pre-existing renal impairment, cardiovascular risk factors, including diabetes mellitus and hypertension, and the use of ritonavir-boosted regimens [22,24–26].

Despite these established risks, there are limited data concerning renal outcomes associated with the use of TDF for PrEP. Adverse renal outcomes have not been reported in a consistent format amongst randomised clinical trials of PrEP. Most randomised trials of TDF-based PrEP report no difference in the rate of adverse renal events between PrEP and placebo arms [5–12]. Renal adverse events reported in randomised controlled trials of PrEP include transient or sustained elevations in serum creatinine concentration, occasionally requiring cessation of the study drug [10,13–16], and one case of transient isolated hypophosphataemia [7]. To the best of the authors knowledge, no cases of overt proximal tubular dysfunction or Fanconi syndrome have been reported in clinical trials of PrEP. The rate of mild elevations in creatinine concentration has ranged from 0–18%, but in most studies was less than 3·5%. Many of these observed differences may be accounted for by differing study settings, population characteristics and duration of follow-up [5–12].

More severe renal impairment, consistent with our definition of a sustained reduction in eGFR to less than 60 ml/min/1·73m^2^, was uncommonly observed in PrEP trials. In the IPERGAY trial, with a median follow-up time of only 9·3 months, two patients (1%) in the TDF-FTC cohort experienced a fall in eGFR to <60 ml/min/1·73m^2^ [13]. In the Bangkok Tenofovir Study, with a total of 9,665 person-years of follow-up, a grade two or greater increase in serum creatinine occurred in five participants (0.4%) in the TDF group and three participants (0.2%) in the placebo group [11]. In our study, with a similar duration of follow-up, the rates were higher; 0·7% in the PrEP cohort and 4·1% in PLWHIV. One study followed participants from the Partners PrEP Study, with a mean duration on study drug of 33 months, and found a small but statistically significant difference in eGFR between participants prescribed TDF/FTC compared with placebo (128ml/min/1·73m^2^ compared to 131ml/min/1·73m^2^, p<0·01) which reversed within four weeks of drug discontinuation, even after three years of PrEP [27]. Randomised controlled trials of PrEP often excluded patients with renal impairment or proteinuria, and in some cases other risk factors for renal disease including viral hepatitis, limiting the generalisability of their experience of adverse renal events to real-world cohorts of patients prescribed PrEP. In our study, half of all patients prescribed ART for HIV infection and a quarter of all patients prescribed PrEP had a baseline eGFR between 60 and 90 ml/min/1·73m^2^ consistent with stage two chronic kidney disease. A meta-analysis of individual participant data from randomised controlled trials and cohort studies, including longitudinal data from 14,368 subjects, demonstrated a 2.43% rate of decline in eGFR to <60 ml/min/1·73m^2^ among TDF-based PrEP users over a medium follow-up time of 10 months [28]. These data suggest a higher rate of incident renal impairment than our study. This may be partly explained by the diversity of participants included in meta-analysis and their individual risk factor profile. Similar to our study, the meta-analysis identified baseline eGFR and increasing age as risk factors for incident renal impairment on PrEP.

Across our entire study population, sustained renal impairment was associated with HIV-positive status, older age and baseline renal impairment. Baseline renal impairment was very common in both cohorts, but significantly more so in the PLWHIV HIV compared with PrEP cohort (50·3% vs. 25·1% in the PLWHIV and PrEP cohorts, respectively). Rates of a positive hepatitis B surface antigen at any time during follow-up were low but significantly higher in the HIV cohort, while evidence of previous exposure to hepatitis C was ten-fold more common in PLWHIV. The estimated prevalence of chronic hepatitis B infection in our cohort lies between the prevalence estimates for Australian-born non-Indigenous people (0·2%) and Australian gay and bisexual men (3%) [29]. Hepatitis B and C infection are associated with glomerular immune-complex deposition and were both associated with increased risk of progression to chronic kidney disease in patients with HIV infection independent of the presence of circulating hepatitis viraemia in the ESPIRT and SMART trials [30]. We did not observe an association between hepatitis B or C and the risk of sustained renal impairment. This may be explained by the widespread access to subsidised treatment for hepatitis B and C in Australia. In a post-hoc analysis of the ESPIRT and SMART trials the difference in risk of progressive kidney disease associated with viral hepatitis became apparent beyond four years of follow-up [30], and as such the effect of hepatitis virus infection on CKD risk may not have been captured in the three years of follow-up in our study.

It is unclear whether medication compliance and drug exposure varied between the PrEP and HIV cohorts in our study. The use of intermittent or on-demand PrEP was not recommended in Australia during the period of this study. It is possible however that drug exposure was lower among the PrEP cohort due to differences in medication compliance. For instance, in 2019 patient-initiated intermittent or on-demand PrEP was reported by 4% and 9.4% of PrEP users in Melbourne and Sydney respectively, Australia’s two most populous cities. Longer periods of PrEP exposure appear to be associated with sustained renal impairment among PrEP users, and TDF-induced renal impairment appears to be reversible with early drug withdrawal amongst patients treated for HIV and PrEP, suggesting a possible protective effect of intermittent drug exposure [27]. Nonetheless, on-demand PrEP was associated with a higher rate of adverse renal outcomes than placebo in the IPERGAY trial [13].

In propensity matched analysis, which included 1,622 participants from each cohort balanced for baseline characteristics including some known risk factors for kidney disease (Table 3), we found residual confounding of risk of sustained renal impairment between the cohorts. After propensity matching, participants in the PrEP cohort still remained at a 52% lower risk of sustained renal impairment than those prescribed ART for HIV infection. The magnitude of this difference in risk implies a significant undefined difference in susceptibility to sustained renal impairment between the cohorts after adjustment for some known risk factors for kidney disease. One possible explanation, and a key limitation of our study, is that our data remain unadjusted for many important risk factors for kidney disease; for example, data were not available for diabetes, hypertension, smoking status or a history of cardiovascular disease, which are all associated with incident CKD in people living with HIV [31]. Another limitation of our studies is that it remains unadjusted for HIV-specific factors that are associated with risk of renal impairment, including a prior AIDS-defining illness, nadir CD4+ count and current CD4+ count. We assumed perfect adherence to the prescribed treatment regimen and some participants may have switched or ceased ART regimens during the follow-up period which would most likely dilute the true risk associated with TDF-based regimens. Another limitation of our analysis is the lack of adjustment for the possible increase in serum creatinine seen among PLWHIV prescribed ART that inhibits tubular secretion of creatinine resulting in a confounding decline in eGFR. Finally, we chose to compare rates of sustained renal impairment between PLWHIV prescribed TDF containing ART with people prescribed TDF for PrEP as these groups are both commonly encountered by ART and PrEP prescribers with our results helping to inform clinical risk assessment. Alternatively, a comparison between a cohort of people taking TDF for PrEP with age and comorbidity matched controls may also be informative. Finally, reporting of eGFR varied throughout the study and from clinic to clinic based on the equation used by pathology laboratories which may be a source of potential error.

In conclusion, we found that participants prescribed TDF-based PrEP had significantly lower rates of sustained renal impairment than PLWHIV prescribed ART, despite a high prevalence of baseline renal impairment. Rates of sustained renal impairment amongst patients prescribed PrEP outside the setting of randomised clinical trials appear to be low. A history of hepatitis C or hepatitis B infection was not associated with an increased risk of renal impairment during a three-year follow-up period. After propensity matching, the participants prescribed ART for HIV infection remained at a significantly higher risk of sustained renal impairment suggesting either unmeasured confounders or potentially a HIV-associated susceptibility to ART nephrotoxicity. People prescribed TDF-based PrEP should have their kidney function monitored according to current clinical practice guidelines. People over age 40 and those with an eGFR <90 ml/min/1.73m^2^ who are prescribed PrEP may benefit from more frequent monitoring of their kidney function. Future studies should aim to collect more comprehensive data on other renal risk factors to determine whether our findings remain significantly worse for HIV-positive individuals compared with individuals taking TDF-based PrEP.

## Supporting information

S1 Appendix Study enrolment. (DOCX)
