# Supplementary material for: Renal impairment associated with tenofovir disoproxil fumarate for antiretroviral therapy and HIV pre-exposure prophylaxis: An observational cohort study
Source: PLoS One. 2023 Feb 24;18(2):e0280339. doi: 10.1371/journal.pone.0280339 (PMC9955644; doi:10.1371/journal.pone.0280339)
Supplement: S1 Appendix — (DOCX) [file pone.0280339.s001.docx]

**Appendix 1.** Study enrolment.

*Participants were excluded if they had a recorded diagnosis of HIV but were prescribed PrEP (n=65), if they were prescribed both PrEP and ART (n=36), or if they were prescribed ART but had no recorded diagnosis of HIV infection (n=6,394).
